# Injectable Hydrogels Based on Pluronic/Water Systems Filled with Alginate Microparticles for Biomedical Applications

**DOI:** 10.3390/ma12071083

**Published:** 2019-04-02

**Authors:** M. T. Cidade, D. J. Ramos, J. Santos, H. Carrelo, N. Calero, J. P. Borges

**Affiliations:** 1 i3N/CENIMAT, Department of Materials Science, Faculty of Sciences and Technology, Universidade, NOVA de Lisboa, Campus de Caparica, 2829-516 Caparica, Portugal; dj.ramos@campus.fct.unl.pt (D.J.R.); h.carrelo@campus.fct.unl.pt (H.C.); 2 Applied Rheology, Colloid Technology, Chemical Engineering Department, University of Sevilla, c/ P. García González, 1, E41012 Sevilla, Spain; jsantosgarcia@us.es (J.S.); nuriacalero@us.es (N.C.)

**Keywords:** Pluronic/water systems, alginate microparticles, composites, injectable gels, rheology, dual cargo delivery systems

## Abstract

A (model) composite system for drug delivery was developed based on a thermoresponsive hydrogel loaded with microparticles. We used Pluronic F127 hydrogel as the continuous phase and alginate microparticles as the dispersed phase of this composite system. It is well known that Pluronic F127 forms a gel when added to water in an appropriate concentration and in a certain temperature range. Pluronic F127 hydrogel may be loaded with drug and injected, in its sol state, to act as a drug delivery system in physiological environment. A rheological characterization allowed the most appropriate concentration of Pluronic F127 (15.5 wt%) and appropriate alginate microparticles contents (5 and 10 wt%) to be determined. Methylene blue (MB) was used as model drug to perform drug release studies in MB loaded Pluronic hydrogel and in MB loaded alginate microparticles/Pluronic hydrogel composite system. The latter showed a significantly slower MB release than the former (10 times), suggesting its potential in the development of dual cargo release systems either for drug delivery or tissue engineering.

## 1. Introduction

Hydrogels consist of three-dimensional crosslinked hydrophilic polymeric networks that absorb and retain large amounts of water or biological fluids while maintaining the structure; they have several unique characteristic features, which include resemblance to tissues’ extra cellular matrix (ECM), support of cell proliferation and migration, controlled release of growth factors, cell and drugs, minimal mechanical irritation to surrounding tissue [1,2].

Hydrogels have been used extensively in the development of smart drug delivery systems. One method of hydrogel preparation is the covalent crosslinking of a precursor solution [3]. Alternatively, physical hydrogels can form when a specific stimulus, like temperature, is applied, or in the presence of ions [4,5].

Thermally responsive hydrogels are particularly interesting because their gelation and changes in swelling can be triggered by temperature change [6,7,8]. In biomedical applications, this can be accomplished through temperature increase from ambient to physiological. These systems allow for in situ hydrogel formation, where a biomaterial can be delivered in solution in a minimally invasive manner and solidify inside the body. Hydrogel formation for many systems happens almost instantaneously once the gelation temperature is reached [9,10].

Stimuli-responsive hydrogels can be loaded with drugs, cells, and growth factors and control their release by changing the gel structure in response to application of the external stimuli [11]. Many physical and chemical stimuli have been applied to induce various responses of the smart hydrogel systems. The physical stimuli include temperature, electric and magnetic fields, light, and pressure, while the main chemical stimuli include pH [2,12]. Smart hydrogels have been used in diverse applications, such as in making artificial muscles [13,14], chemical valves [15], and self-regulated drug delivery systems [16,17,18].

Hydrogels have been used in injectable system which will result in a minimal invasive surgical procedure with decreased patient morbidity, a lower risk of infection and the ability to fill irregular shaped defects avoiding the need for patient specific prefabrication [19,20,21].

Pluronics (PEOx-PPOy-PEOx) are non-ionic triblock copolymers composed of two hydrophilic side chains of poly (ethylene oxide), PEO, and a central hydrophobic chain of poly (propylene oxide), PPO. They are also known as poloxamers and are non-toxic, biodegradable and FDA approved [22]. There are an extensive variety of Pluronics in the market named by its molecular weight and the ratio between hydrophobic and hydrophilic units. They have many applications in different fields such as cosmetics, pharmaceuticals and food emulsions [23,24,25,26] due to their self-assembly properties, interfacial properties and their ability to form thermoreversible micelles and gels [27,28,29].

Pluronic F127^®^ is one of the most used in regenerative medicine. One of its main characteristics is that it forms a gel at relatively low concentrations and temperatures close to room temperature [30,31], which is very interesting for biomedical applications. In addition, it is widely used due to its simple phase diagram with water compared to other Pluronics [31]. Because of these features, the binary systems Pluronic F127/water have been used in clinical applications such as drug and gene delivery [32] and cell separation [33].

In this paper we report the effect of the addition of alginate microparticles to the Pluronic F127/water system, in the rheological behavior of the composites, in view of its application as injectable hydrogel that may present two different rates of drug release, one from the gel itself and another one from the added microparticles. In addition, the kinetics of drug release in the hydrogel, and in the alginate microparticles/Pluronic hydrogel composites was also determined.

## 2. Experimental

### 2.1. Materials and Sample Preparation

The triblock copolymer Pluronic^®^F127 (Sigma-Aldrich, Darmstadt, Germany) was used as received. The aqueous solutions of F127 were prepared by *cold method*: required amounts of deionized water and copolymer was weighted and mixed in a vial, sealed and kept in a refrigerator until a homogeneous solution was formed (typically 1 or 2 days). Solutions of different Pluronic concentrations, 12.5, 15, 15.5, 17.5, 20, and 22.5 wt%, were prepared. For the preparation of the microparticles a 1 wt% aqueous solution of Na-alginate was used (Alginic Acid Sodium Salt, Panreac AppliChem, Darmstadt, Germany). The solution was extruded dropwise into a 5 wt% CaCl_2_ aqueous solution. Upon contact with the crosslinking solution (CaCl_2_) almost spherical-shaped particles instantaneously formed and could harden overnight. The size was controlled by regulating the flow rate using a syringe pump (KDS) and by applying a coaxial air stream (Encapsulation Unit Nisco, Zurich, Switzerland, model Var J1). After completion of the gelling period the microspheres were recovered, rinsed with water to remove CaCl_2_ in excess and dried overnight in a vacuum oven at 30 °C. For a selected concentration of Pluronic/water system, different amounts of alginate microparticles were added: 5, 10, and 15 wt%. Methylene blue (MB, high purity, Alfa Aesar, Karlsruhe, Germany) was selected as a water-soluble model drug. Blank alginate microparticles (572 mg) were suspended in 30 mL of MB solution (25 mM). The suspension was stirred for 24 h at room temperature and then lyophilized. MB-loaded microparticles were then dried and stored in a desiccator for further evaluation.

### 2.2. Particle Size Distribution

The Sauter diameter (*D*[3,2]) and volumetric mean diameter (*D*[4,3]) of the alginate microparticles was determined by Laser Diffraction in a Mastersizer 2000 (Malvern, Worcestershire, UK). To do so, a small portion of alginate microparticles was suspended in water before loading into the apparatus.

### 2.3. Rheological Characterization

The rheological characterization was performed in an AR2000 rotational rheometer (TA Instruments, New Castle, DE, USA), using a plate/plate geometry (60 mm diameter) and a gap of 1 mm in case of Pluronic/water system and 2 mm in case of the composites. A solvent trap was used to prevent water evaporation. Steady state and oscillatory shear measurements were performed at different temperatures and in function of temperature. The measurements were made in triplicate.

### 2.4. Drug Release

In vitro release assays were performed using a home-made membrane diffusion system with a permeation area around 3.14 cm^2^, with artificial membranes acting as a barrier. First, a solution with 25 mM of MB in Pluronic and a composite system consisting of a suspension of microparticles loaded with MB in Pluronic (10% (wt/v) in Pluronic) were prepared. The donor compartment was filled with 5 mL of Pluronic/MB solution or 5 mL of Pluronic/MB-loaded microparticles. The receptor compartment was filled with 55 mL of PBS (Phosphate Buffer Saline) solution. After filling the diffusion system was kept at 37 °C. Aliquots from the receptor compartment (at regular periods of time intervals for both assays) were withdrawn (30 mL) and analyzed by UV-vis spectrophotometry (T90+ UV/VIS Spectrometer, PG Instruments Ltd., Leicestershire, UK). The amount withdrawal was replaced with fresh PBS. The measurements were made in triplicate.

## 3. Results and Discussion

### 3.1. Particle size distribution (PSD)

The particles present a monomodal distribution with the peak centered at about 417 µm. The mean particle diameter is expressed as Sauter diameter (*D*[3,2]) and volumetric mean diameter (*D*[4,3]), given by Equation (1).

(1) $$D\left[M,N\right]={\left[\frac{\int D^{M}\;n\left(D\right)dD}{\int D^{N}\;n\left(D\right)dD}\right]}^{\frac{1}{M-N}}$$

The results obtained were: *D*[3,2] = 379 µm; *D*[4,3] = 401 µm.

### 3.2. Rheological Characterization

#### 3.2.1. Pluronic/Water Systems

Pluronic F127, like other Pluronics, are amphiphilic polymers that undergo micellization in water, through the hydrophobic self-aggregation, when a critical micellization concentration (CMC), at room temperature, or a critical micellization temperature, for a certain concentration, are reached. The assembling of micelles, due to the decrease of intermicellar distance, forms a gel network, through entanglements, at a critical temperature, the gelation temperature. Two mechanisms were proposed for the decrease of intermicellar distance [34]. In the first one, the micellar sizes remain the same but new micelles are formed during heating, approaching each other, while in the second one, the number of micelles remains unchanged but their size is increased with temperature, which also brings them closer to each other.

**Figure 1:**
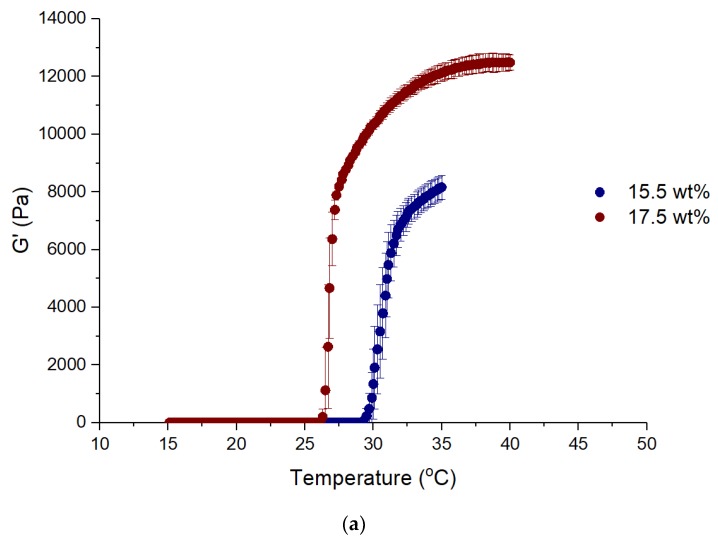

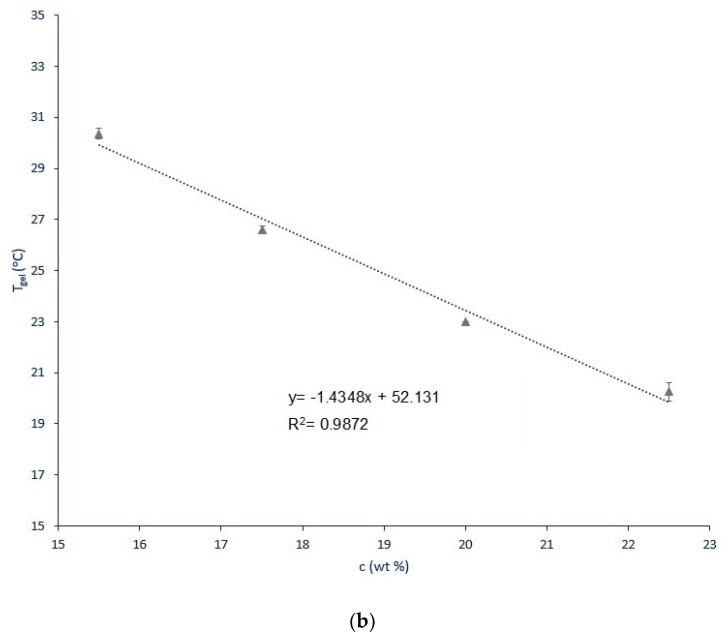
Elastic modulus as a function of temperature for 15.5 and 17.5 wt% Pluronic concentrations (**a**) and gelation temperature as a function of Pluronic concentration (**b**). Values are represented as mean ± standard deviation (*n* = 3).

**Figure 2:**
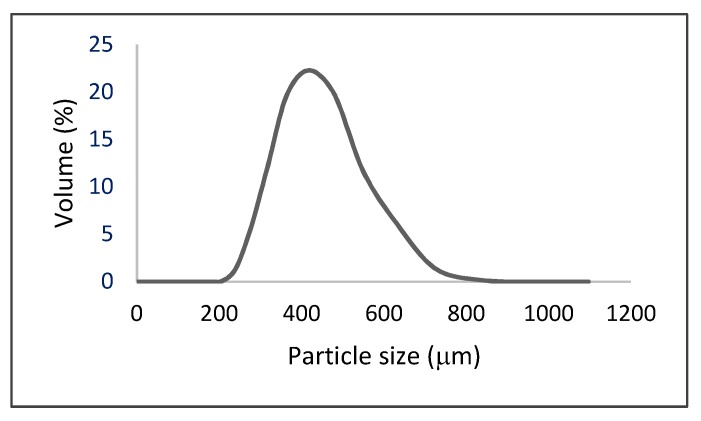
Particle size distribution (PSD) of prepared alginate microparticles.

Figure 1a presents the sol-gel transition followed by oscillatory shear measurements for two Pluronic concentrations, while Figure 1b shows the gelation temperature (T_gel_) for all the concentrations studied, except for the less concentrated ones (below 15.5 wt%) that did not show any gelation. T_gel_ was determined from the crossover between G′ and G′′. For sake of clarity only G′ is presented in Figure 2. As can be seen, at the sol-gel transition the standard deviations are high, however, the determined values of T_gel_ are accurate since their standard deviations are small (Figure 1b). Similar gelation temperatures were already determined [35,36].

The gelation temperature decreases linearly with copolymer concentration as shown in Figure 2. This is a common finding, for either Pluronic F127 [35,36] or for other Pluronics [34,37].

**Figure 3:**
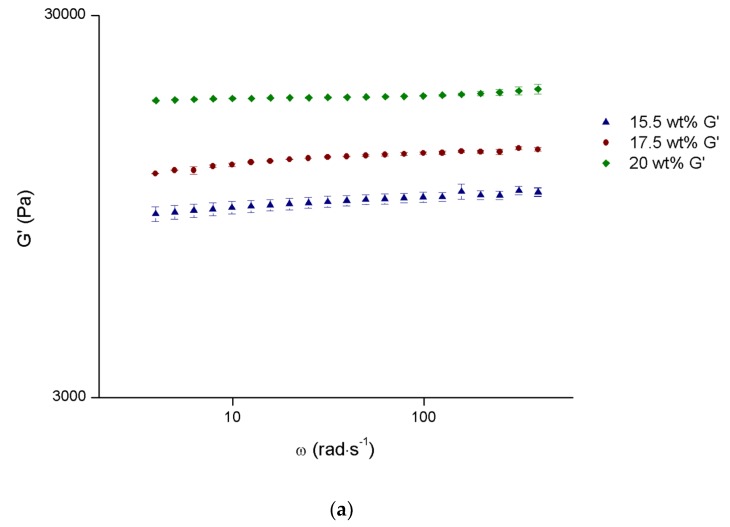

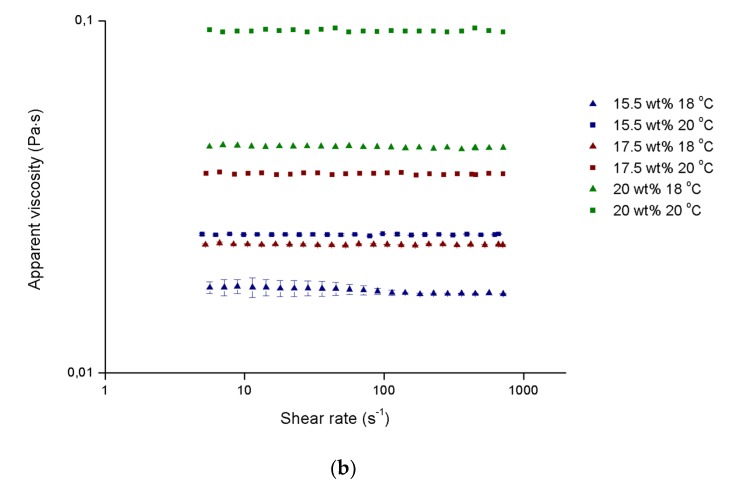
G′ as a function of angular frequency at 37 °C (**a**) and viscosity curves at 18 and 20 °C (**b**) for selected Pluronic concentrations. Values are represented as mean ± standard deviation (*n* = 3).

Figure 3a shows the results of the frequency dependence of elastic (G′) modulus, in the LVR, at 37 °C (body temperature) for selected concentrations, and the viscosity curves at 18 and 20 °C (around surgery room temperature) are presented in Figure 3b, for the same concentrations. Figure 3a shows that the elastic modulus is almost independent of the angular frequency, a characteristic of the gel state. The same behavior was already reported [38]. It also shows that the elastic modulus increases with concentration of Pluronic at 37 °C (gel state), as expected, and also found by Zhao et al. [39].

Figure 3b shows that the apparent viscosities at relatively low temperatures (sol state) increase with polymer concentration, as expected in a polymer solution. An increase of viscosity is also observed when increasing the temperature from 18 to 20 °C, which is due to the closest approximation to T_gel_ and the decrease of the intermicellar distance.

Considering the intended application, the optimal system would be the one presenting a T_gel_ as closest as body temperature as possible, the highest elastic modulus at body temperature and the smallest apparent viscosity during the injection process. The 15.5 wt% system fulfills two of those requirements, and its G′ values at 37 °C, despite being the smallest ones, are high enough to keep it in place in body cavity, so, this concentration was chosen for basis of the composite system.

#### 3.2.2. Pluronic/Water System Filled with Alginate Microparticles

**Figure 4:**
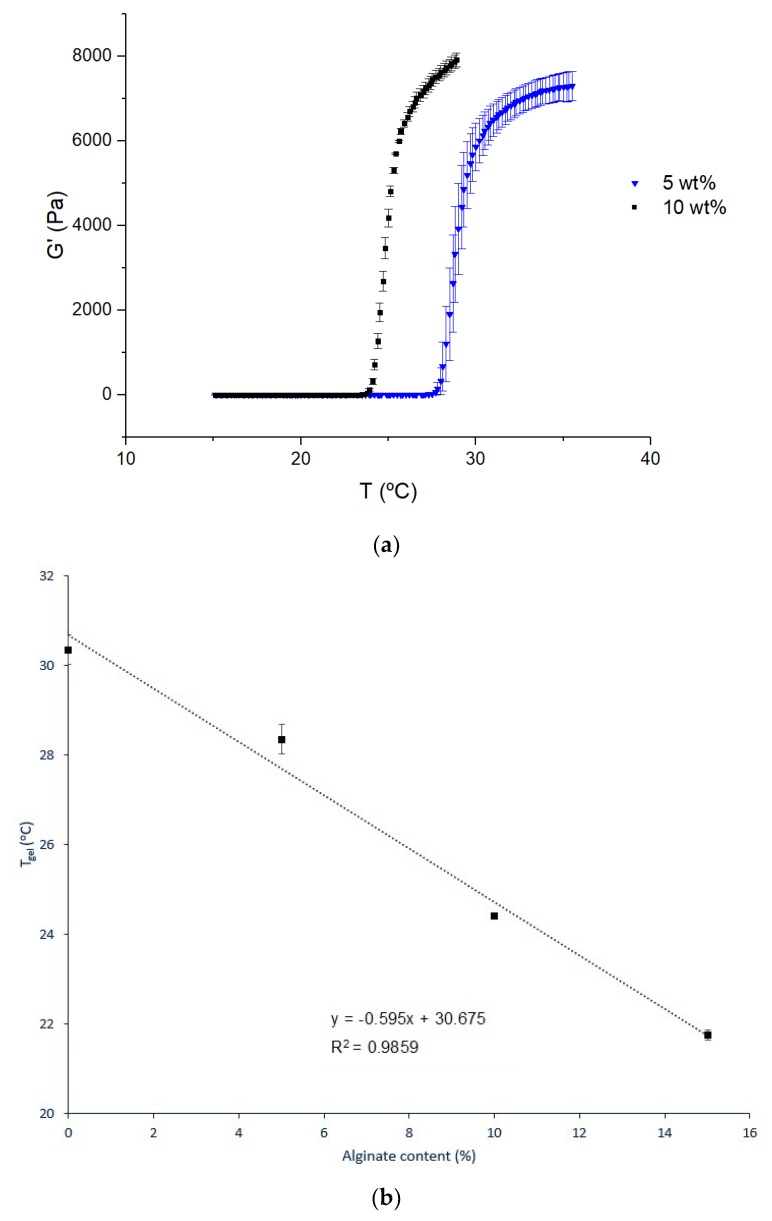
Elastic modulus in function of temperature for 2 contents of alginate microparticles in a 15.5 wt% Pluronic/water system (**a**) and gelation temperature as a function of alginate microparticles content (**b**). Values are represented as mean ± standard deviation (*n* = 3).

Figure 4a presents the sol-gel transition followed by oscillatory shear measurements for two different contents of alginate microparticles, as examples, while Figure 4b shows the dependence of the sol-gel transition temperature (T_gel_) with the content of alginate microparticles. As observed, the higher the alginate microparticles content, the smallest T_gel_. This decrease in T_gel_ may be explained by hydrogen bonds between the carboxyl groups of alginate and ether groups of Pluronic, which may lead to the formation of a three-dimensional network at temperatures below the ones at which the micelles of Pluronic F127 entangle to form the gel [40].

Once again, despite the fact that, at the sol-gel transition, the standard deviations are high (especially for 5 wt% alginate microparticles content, as shown in Figure 3a), the determined values of T_gel_ are accurate since their standard deviations are small (Figure 3b).

**Figure 5:**
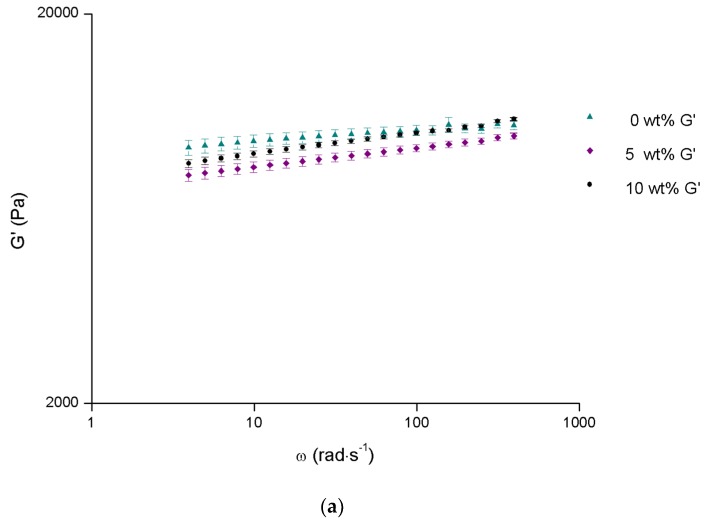

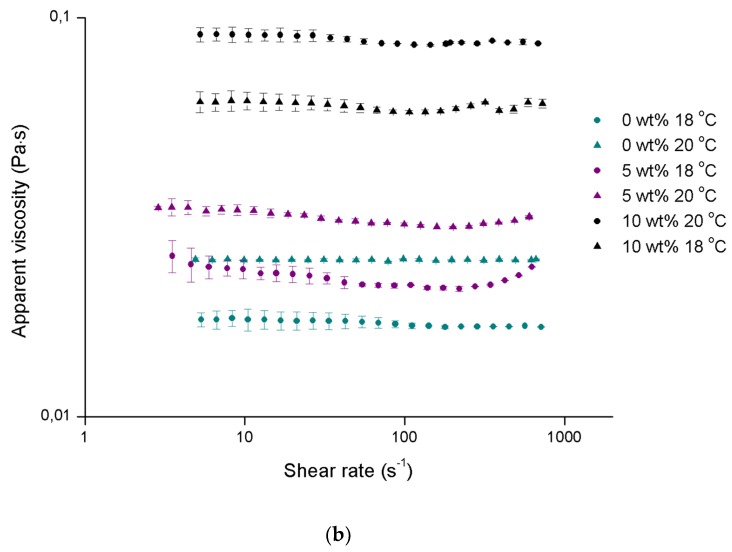
G′ as a function of angular frequency (**a**) and viscosity curves (**b**) for different contents of alginate microparticles in a 15.5 wt% Pluronic/water system. Values are represented as mean ± standard deviation (*n* = 3).

Figure 5a shows the results of the frequency dependence of elastic (G′) modulus at 37 °C (body temperature) for different contents of alginate microparticles, and Figure 5b presents the viscosity curves at 18 and 20 °C (around surgery room temperature) for the same composites. Note that the 15 wt% alginate microparticles results are not presented since the gelation temperature is already too low for the intended application. In fact, being the gelation temperature just slightly higher than the temperature of the injected solution, it will immediately jellify when entering the body, not leaving time for the complete injection.

As seen in Figure 5a, the elastic modulus is almost independent of alginate microparticles content, the differences lying within the experimental error. This result shows that the elastic behavior is dictated by the micellar structure of Pluronic F127. On the other side, the increase of the alginate microparticles content increases the shear apparent viscosity (Figure 5b), as expected when microparticles are added to a viscous continuum phase, and, once again, an increase in temperature leads to higher viscosity, due to the decrease of intermicellar distance with temperature.

**Figure 6:**
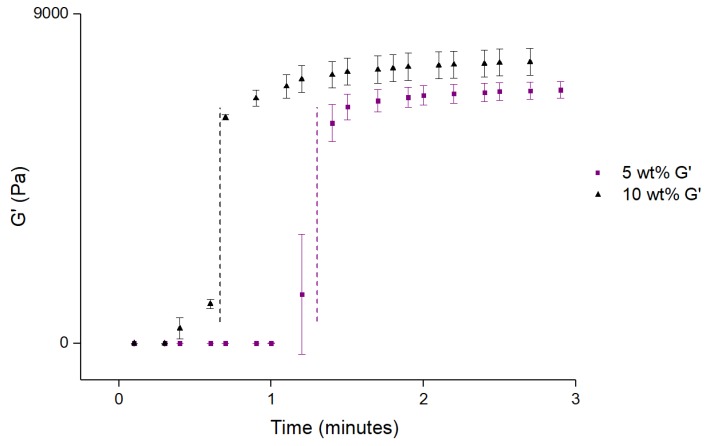
G′ and G′′ (f = 1 Hz) as a function of time after a sudden increase in temperature from 15 to 37 °C for 5 and 10 wt% alginate microparticles content in a 15.5 wt% Pluronic/water system. The vertical discontinuous lines represent the maximum time allowed for the injection procedure. Values are represented as mean ± standard deviation (*n* = 3).

To study the time available for the injection, time sweep tests were performed. The solution was kept at 15 °C before the measurement took place, and then the viscoelastic modulus was measured with a set temperature of 37 °C. The time elapsed between the start of the measurements and the moment a crossover between G′ and G′′ took place (which was obviously due to the increase in temperature during the measurements) was considered the time available for the injection. Figure 6 presents the results obtained for 5 and 10 wt% alginate microparticles content, the ones of interest, as concluded from previous results.

The analysis of Figure 6 allows for the determination of the limiting time for the injection procedure, the time at which G′ increases abruptly. The increase of alginate microparticles content from 5 to 10 wt%, decreases the limiting time from around 80 s to a little less than 40 s. The maximum content of alginate microparticles will then be conditioned by the size and location of injury, that will dictate the time needed for the injection procedure.

### 3.3. Drug Release

**Figure 7:**
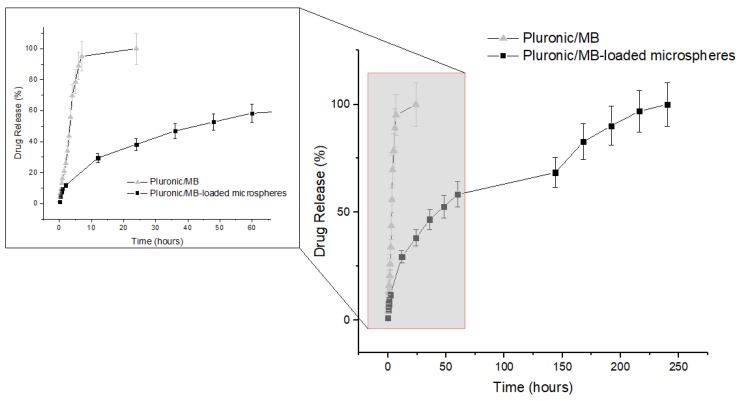
Release profiles of Methylene Blue in PBS for Pluronic/MB and Pluronic/MB-loaded microspheres systems. Values are represented as mean ± standard deviation (*n* = 3).

Figure 7 shows the release profiles of 15.5 wt% Pluronic hydrogel/10 % (wt/v) MB-loaded microparticles (composite) and 15.5 wt% Pluronic hydrogel/MB systems at body temperature. It can be observed that in the composite system MB has a much slower release. After 24 h MB was completely released from the 15.5 wt% Pluronic aqueous solution/MB system while for the composite system only 38.2% of MB was released in the same period and complete release of MB only happened after 240 h. This delayed release obtained for the composite system is due to different release kinetics of MB from the microspheres and from the Pluronic hydrogel. Drug release experiments here presented suggest that 15.5 wt% Pluronic hydrogel/10% (wt/v) MB-loaded microspheres can be used in dual drug release systems. Release experiments using different drugs in the alginate microspheres and in the Pluronic hydrogel must be performed to further characterize these dual systems. Combined therapy with drugs of different therapeutic effects using Dual Drug Delivery Systems (DDDS) is an effective way to control the release behavior of each drug independently. Combined delivery of anticancer drugs with different mechanisms of action allows overcoming undesirable toxicity and other side effects and to circumvent multidrug resistance [41]. Many DDDS reported in the literature are based on hydrogel/polymeric micelle composites [41,42,43,44]. These dual systems have also been used for controlled delivery in hard tissue engineering [45,46], where different drugs or a combination of drugs and cells or growth factors were used.

Fitting of the drug release data was performed using a freely available add-in program called DDSolver, developed by Zhang et al. [47]. The best model was chosen considering the one with the highest adjusted coefficient of determination, which can be determined by Equation (2) [48]:

(2) $$R_{adj}^{2}=1-\frac{n-1}{n-p}\left(1-R^{2}\right)$$

For both systems the Korsmeyer–Peppas model was the one that best fitted the MB release data, presenting a $R_{adj}^{2}$ value of 0.98.

Korsmeyer–Peppas model is described by Equation (3):

(3) $$Q_{t}=kt^{n}$$

where $Q_{t}$ is the fraction of drug released at time t and *k* is the release rate. The exponent *n* gives information about the release mechanism. If *n* ≤ 0.43 it is a Fickian diffusion and if *n* = 0.85 it is a case II transport, being related to polymer matrix relaxation and swelling. If 0.43 < *n* < 0.85 it corresponds to an anomalous transport, resultant form the combination of both mechanisms. A special case, when *n* > 0.85, corresponds to a super case II transport. For the 15.5 wt% Pluronic aqueous solution/MB, a value of *n* = 1.00 was obtained, corresponding to a super case II transport mechanism. In this case, the release mechanism is dependent on the relaxation of the polymeric chains. For the composite system the value of *n* was approximately 0.46. This value is close to the 0.43 limit, which means that the release mechanism is predominantly related to the Fickian diffusion of MB through the system [48,49]. In the composite systems the MB is firstly released from the microspheres into the Pluronic hydrogel and afterwards will diffuse through it. Total release of MB from the alginate microspheres can only be observed after their complete swelling/dissolution and, for this reason, an extended release is observed in the composite system (see Figure 6).

## 4. Conclusions

The Pluronic F127^®^/water system with different polymer concentrations were characterized rheologically, showing that 15.5 wt% polymer concentration is an adequate concentration for use as injectable gel. The effect of the addition of different amounts of alginate microparticles to the 15.5 wt% Pluronic/water system in the rheological behavior was analyzed.

The results obtained showed that the addition of the microparticles are not determinant in the value of the elastic modulus at body temperature, however, it increases the viscosity of the solutions and decreases the gelation temperature, which limits the percentage of microparticles to be added. The time that takes, after a sudden increase in temperature from 15 to 37 °C, for the gelation to occur, also depends on the concentration of alginate microparticles, decreasing with its increase. In fact, the increase of alginate microparticles content from 5 to 10 wt%, decreases the limiting time available for the injection procedure from around 80 s to a little less than 40 s. The maximum content of alginate microparticles will then be conditioned by the size and location of injury, that will dictate the time needed for the procedure.

In order to perform drug release studies 10% (wt/v) alginate microparticles addition, relative to the weight of hydrogel, was chosen. Drug release results showed that the incorporation of MB-loaded alginate microparticles in 15.5 wt% Pluronic hydrogel delayed 10 times the release of MB, when compared with MB/Pluronic hydrogel system alone, demonstrating its potential in the development of dual release systems either for drug delivery or tissue engineering. Other release studies using different drugs (or a combination of drugs and cells or growth factors) in the alginate microparticles and in the Pluronic hydrogel must be performed considering the intended application Further stability studies regarding the integrity of these dual systems must be performed, which are also dependent on the drugs or drug/cell/growth factors combinations used in their production.
